# Economic evaluation of the Liverpool heart failure virtual ward model

**DOI:** 10.1093/ehjqcco/qcae095

**Published:** 2024-11-13

**Authors:** Debar Rasoul, Ipsita Chattopadhyay, Tony Mayer, Jenni West, Hadleigh Stollar, Casey Black, Emeka Oguguo, Rosie Kaur, Rachael MacDonald, Jessica Pocock, Barbara Uzdzinska, Bethany Umpleby, Nick Hex, Gregory Yoke Hong Lip, Rajiv Sankaranarayanan

**Affiliations:** Liverpool University Hospitals NHS Foundation Trust, Aintree Hospital, Liverpool, Lower Lane, L9 7AL, UK; Liverpool Centre for Cardiovascular Science at University of Liverpool, Liverpool John Moores University and Liverpool Heart & Chest Hospital, Brownlow Hill, L69 7TX, UK; MHLDC Provider Collaborative, Cheshire & Merseyside, UK; MHLDC Provider Collaborative, Cheshire & Merseyside, UK; Health Innovation NW Coast, Liverpool, Keckwick Lane, WA4 4AB, UK; Health Innovation NW Coast, Liverpool, Keckwick Lane, WA4 4AB, UK; Health Innovation NW Coast, Liverpool, Keckwick Lane, WA4 4AB, UK; Liverpool University Hospitals NHS Foundation Trust, Aintree Hospital, Liverpool, Lower Lane, L9 7AL, UK; Mersey Care NHS Foundation Trust, Liverpool, Prescot, L34 1PJ, UK; York Health Economics Consortium, University of York, Innovation Way, YO10 5NQ UK; York Health Economics Consortium, University of York, Innovation Way, YO10 5NQ UK; York Health Economics Consortium, University of York, Innovation Way, YO10 5NQ UK; York Health Economics Consortium, University of York, Innovation Way, YO10 5NQ UK; York Health Economics Consortium, University of York, Innovation Way, YO10 5NQ UK; Liverpool Centre for Cardiovascular Science at University of Liverpool, Liverpool John Moores University and Liverpool Heart & Chest Hospital, Brownlow Hill, L69 7TX, UK; Danish Center for Health Services Research, Department of Clinical Medicine, Aalborg University, Aalborg, Denmark; Liverpool University Hospitals NHS Foundation Trust, Aintree Hospital, Liverpool, Lower Lane, L9 7AL, UK; Liverpool Centre for Cardiovascular Science at University of Liverpool, Liverpool John Moores University and Liverpool Heart & Chest Hospital, Brownlow Hill, L69 7TX, UK; GIRFT, NHS, England

**Keywords:** Heart failure, Virtual Ward, Hospital at home, Remote monitoring, Economic analysis

## Abstract

**Background:**

A virtual ward (VW) supports patients who would otherwise need hospitalization by providing acute care, remote monitoring, investigations, and treatment at home. By March 2024, the VW programme had treated 10 950 patients across six speciality VWs, including heart failure (HF). This evaluation presents the economic assessment of the Liverpool HF VW.

**Method and results:**

A comprehensive economic cost comparison model was developed by the York Health Economics Consortium (University of York) to compare the costs of the VW to standard hospital inpatient care [standard care (SC)]. The model included direct VW costs and additional costs across the care pathway. Costs and resource use for 648 patients admitted to the HF VW were calculated for 30 days post-discharge and total cohort costs were extrapolated to a full year. Primary outcomes included costs related to length of stay, readmissions, and NHS 111 contact. The total cost for the HF VW pathway, including set-up costs, was £467 524. This results in an incremental net cost benefit of £735 512 compared with the total SC cost of £1 203 036, indicating a substantial net cost benefit of £1135 per patient per episode (PPPE). This advantage remains despite initial setup expenses and ongoing costs such as home visits, virtual consultations, point-of-care testing, and home monitoring equipment.

**Conclusion:**

Our HF VW model offers a substantial net cost benefit, driven by reduced hospital stays, fewer emergency department visits, and lower readmission rates. The study highlights the importance of considering system-wide impacts and continuous monitoring of VWs as they develop.

## Graphical Abstract

**Graphical Abstract:**
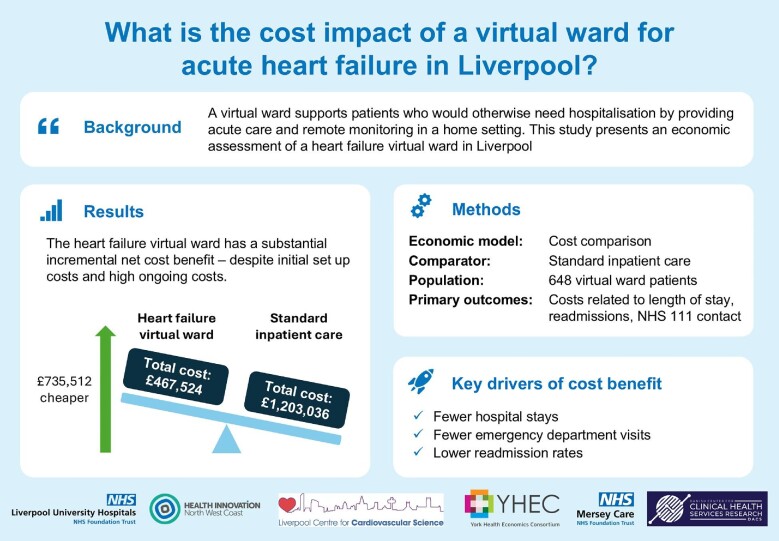

Key Learning Points What is already known

- Virtual wards can serve an alternative to hospitalization or enable early supported discharge for suitable patients.
- Patient, carer, and family attitudes as well as feedback regarding virtual wards is positive.

What this study adds

- Showcases a specialist acute heart failure virtual ward model which provides a net cost benefit.
- Net cost benefit is driven primarily by reduced initial length of stay, reduced attendances to emergency departments and reduced readmission to hospital.
- Results of this study coupled with improved clinical outcomes indicates that a specialist heart failure virtual ward can be cost-effective.

## Introduction

The ‘NHS @Home’^1^ model was introduced as one of the eight components of the ‘Increasing Operational Resilience in Urgent and Emergency care … ’ reforms to address the anticipated winter pressures on the back of a recent COVID-19 pandemic and an unprecedented heatwave in the United Kingdom in August 2022.^2^ The core objective included extra funding and rolling out of ‘virtual wards’ across the country, enabling patients who would otherwise be in hospitals to receive support at home through an increase in capacity by 2500 ‘virtual beds’. Additional goals included an emphasis on patient education and self-management with a vision to allow faster access to more appropriate and targeted care, without the need for patients having to attend emergency care departments or access primary care and NHS 111.

A virtual ward (VW) (also known as ‘hospital at home’) supports patients who would otherwise require hospitalization by providing acute care, remote monitoring, investigations, and treatment within their home or usual place of residence. This includes admission avoidance (AA) or ‘step-up’, where interventions prevent acute hospital ward admissions, and early supported discharge (ESD) or ‘step-down’, which allows patients to leave the hospital earlier than usual by providing necessary interventions at home. The hospital at home (H@H) model integrates in-person care at the patient's residence in combination with remote oversight and monitoring to deliver a care assessment, investigations, or acute level intervention such as IV therapies and prognostic heart failure (HF) medication optimization. VW and H@H programs do not necessitate the use of technology but can be enhanced and delivered as ‘tech-enabled’ programs by utilizing monitoring apps, technology platforms, patient wearables, and medical devices like pulse oximeters to aid in patient monitoring and care delivery.

Over 920 000 patients in the UK live with HF,^3^ with an estimated 200 000 new diagnoses each year.^4^ The impact of HF on hospital admissions is significant, with over 100 000 admissions to hospitals in the United Kingdom,^5^ accounting for 1 million inpatient bed days,^3^ 2% of total NHS inpatient bed days, and 5% all unplanned emergency medical admissions to hospital.^3^ Hospital admissions attributed to HF are projected to rise by 50% over the next 25 years^3^ due to the incidence of HF increasing despite advancements in healthcare, attributed to better survival rates among patients with ischaemic heart disease, enhanced HF therapies, and an ageing population.^4^

Over the last decade, we have utilized telehealth remote monitoring in Cheshire and Merseyside (C&M) for long-term conditions such as HF, chronic obstructive pulmonary disease (COPD), and diabetes, and this model has demonstrated a reduction in emergency admission.^6^ By March 2024, the VW programme in Liverpool had treated 10 950 patients across six speciality VWs—frailty, acute respiratory infection (ARI), palliative care, cancer, paediatrics, and HF—across nine trusts in our integrated care systems authority area.

A ‘Benefit Realisation for Virtual Wards’ project was established to evaluate both quantitative and qualitative outcomes and to also conduct an economic analysis of the VW initiative through a partnership involving the regional healthcare board, innovation agency, research organization, and academic institution. The C&M Integrated Care Board (ICB) formed a partnership with Health Innovation North West Coast (HINWC) and National Institute for Health and Care Research (NIHR) Applied Research Collaborative (ARC) North West Coast to evaluate the programme of work around VWs and commissioned the economic analysis to York Health Economics Consortium (YHEC) at the University of York. In this study we present the economic analysis of the HF VW in our region.

## Methods

### Heart failure virtual ward

For background, referrals to the acute HF VW at Liverpool University Hospitals NHS Foundation Trust consisted of two main categories: AA (step-up) and ESD (step-down). AA includes referrals from the accident and emergency department (AED) and ambulatory units of three large university teaching hospitals in Liverpool (Aintree University Hospital, Royal Liverpool University Hospital, and Liverpool Heart and Chest Hospital), outpatient clinics, and community HF specialist nurses from teams in and around Liverpool (Merseycare NHS Foundation Trust, Liverpool Community Heart Failure Team, Knowsley Community Heart Failure Team, and West Lancashire Community Heart Failure Team), as well as from primary care. ESD refers to referrals from hospital wards within the three hospitals in Liverpool.

The capacity of the VW is 25 patients, although this is flexible and based on demand. A dedicated HF specialist nurse (‘case-finder’) is available from 8 a.m. to 8 p.m., 7 days a week, to receive referrals and assess patient suitability for HFVW. Suitable patients were ‘onboarded’ onto the HFVW remote monitoring digital health platform (DOC@HOME®), and equipment was delivered for daily monitoring, including weighing scales for daily weights, a blood pressure monitor, a pulse oximeter for oxygen saturations thrice a day, a single-lead electrocardiogram, and a wearable device for step count. Patients also received a daily symptom questionnaire.

Patients received daily phone calls from telehealth nurses and face-to-face clinical assessments when receiving IV diuretics, as well as when required through the integration of hospital and community HF specialist nursing teams. Patients with deteriorating clinical symptoms or signs who required prompt clinical assessment could be reviewed at home by a dedicated HF specialist nurse, employed to expand the HFVW team and to allow for home reviews between 8 a.m. and 8 p.m., 7 days a week (‘rapid response HF specialist nurse’). HFVW patients were also provided with access to a mobile phone application (Aintree Heart Failure Passport) to improve patient education, self-care behaviour, and prompts for medication adherence and appointments.

A daily HFVW round was conducted by a HF consultant cardiologist seven days a week, with clinical cover provided from 8 a.m. to 8 p.m., 7 days a week. Patients with ‘red-flag symptoms’ outside HFVW working hours (8 p.m. to 8 a.m.) were advised to contact paramedics and were supported for clinical advice by the hospital on-call cardiology team. Patients received intravenous furosemide administered as a bolus^7^ (no faster than 4 mg/min) using an elastomeric pump in their home (if Rockwood Clinical Frailty Score was >6) or in a HF specialist-delivered ambulatory HF unit. We also used point-of-care (POC) blood tests (iSTAT) for renal function testing and POC echocardiography using the VscanTM to obtain left ventricular systolic function assessments and to rule out significant valve problems. As patients included both *de novo* diagnosis as well as already known diagnosis of HF, this aided prompt initiation of prognostic therapies. Remote electronic prescribing enabled rapid initiation and optimization of prognostic HF therapies by utilizing our previously established HF specialist pharmacist to also conduct HFVW medication optimization clinics.

### Economic model

**Figure 1:**
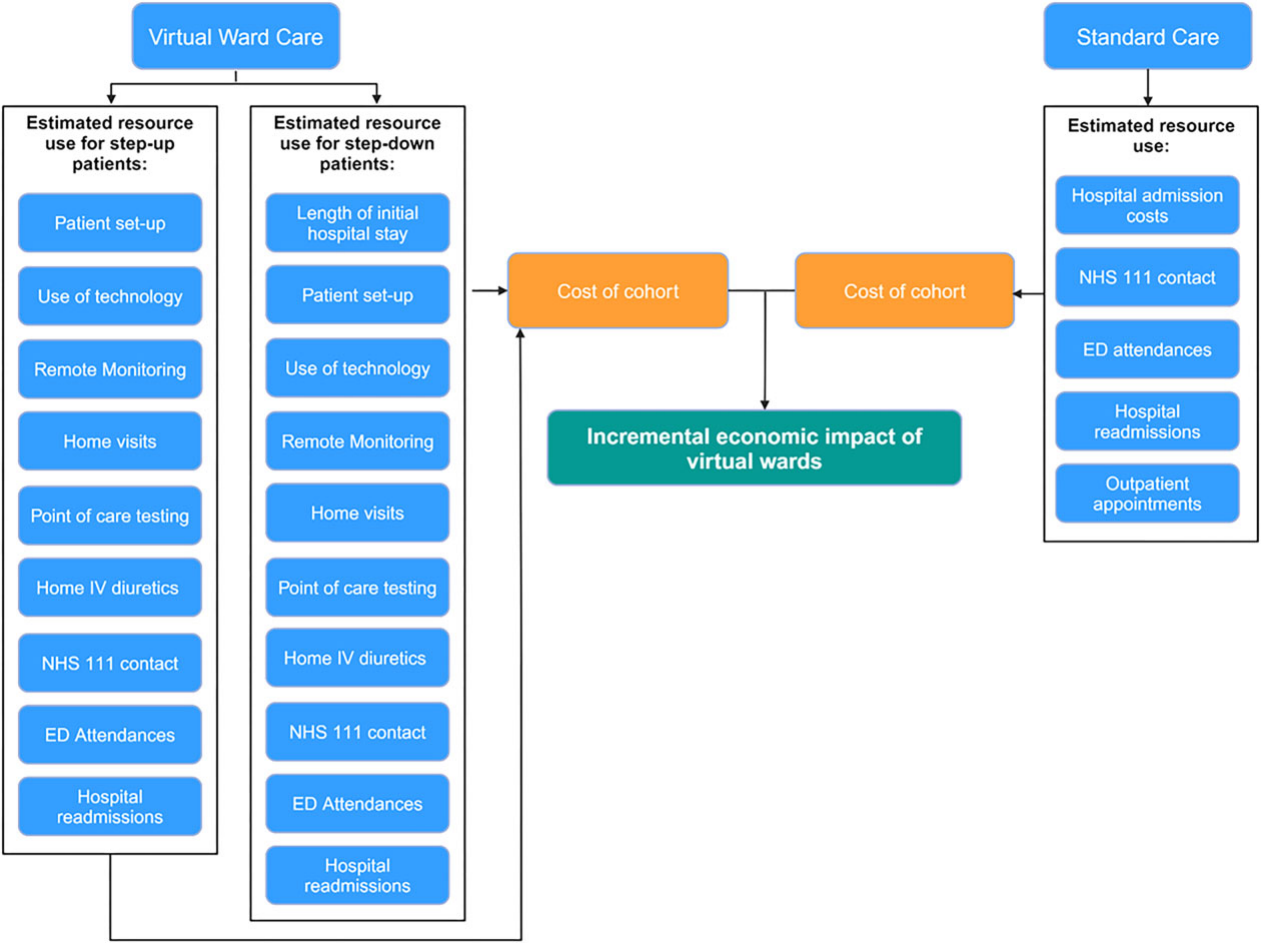
Economic model structure.

A comprehensive economic cost comparison model was developed to evaluate the costs and benefits of VWs to ascertain if the model is cost-incurring or cost-saving in comparison to standard hospital inpatient care. In this paper, we present the economic analysis of the Liverpool HF VW and this model encompasses the entire care pathway of patients admitted to a VW, ensuring that all relevant healthcare system impacts and costs associated with VWs are considered (see *Figure 1*). It includes direct costs incurred by the VW as well as additional costs across the care pathway related to the intervention. By attaching costs to the resource use of patients on VWs and comparing this with the resource use under standard care (SC), the model provides an understanding of the *incremental cost* associated with the VW intervention. SC denotes the treatment patients would typically receive within a hospital setting in the absence of the existence of a VW. The incremental cost per patient shows the cost difference between providing care in the VW vs. SC.

This methodology diverges from traditional ‘management accounting’ approaches, which might simply divide the VW budget by activity. From a health economic standpoint, such an approach presents challenges as it fails to consider resource utilization appropriately. This is crucial because resource usage may vary over time, independent of a VW budget, influenced for instance by the acuity levels of patients admitted to VWs.

Six months' worth of data was extrapolated to a full year to encompass the costs and resource use associated with all patients admitted to the VW. For each patient admitted to the VW, costs and resource use relating to that admission are calculated over 30 days from discharge, and it is assumed any resource use beyond this time is not related to the VW admission. The ‘30 days from discharge’ criterion was used as this is a standard measure in the NHS and health economics to measure outcomes related to an admission. Additionally, the NICE early value assessment of VWs for ARIs considers 30 days sufficient to capture all outcomes related to the intervention and condition.^8^

The key outcomes included within the model include length of stay costs, readmission rates, and cost within 30 days and contact with ‘NHS 111’. Where available, real-world data from our cohorts have been used. Where projection data or costings sourced outside our cohort have been used, it is clearly labelled as such. Resource use costs for these services were obtained from the Personal Social Services Research Unit (PSSRU)^9^ and the 2021/22 NHS Cost Collection,^10^ as these represent the actual costs associated with these resources, in contrast to tariff payments.

Annual costs for the ICB include costs related to remotely monitoring patients (telehealth and Docobo^®^ costs), pharmacy, clinical leadership, staffing, and programme management costs. Communications is a one-off set up cost in the model which was split equally across the VWs and their care pathways, except for the costs of remotely monitoring patients which is a separate cost. Telehealth costs pertain to the Merseycare remote monitoring hub and Docobo^®^ costs are for the equipment patients use when admitted to the HF VW.

The primary aims of VWs are to prevent avoidable admissions into hospital and to support early discharge out of hospital. Hospital admission costs were sourced from the 2021/22 NHS Cost Collection.^10^ The cost for a hospital admission with HF, calculated using non-elective (NEL) short and long stay inpatient stay reference costs, is £2587^10^ per patient per inpatient admission. This cost is based on health resource group (HRG) codes that include all codes for HF or Shock.

Since most costs related to a hospital admission occur at the beginning of an inpatient stay,^11^ the cost saving associated with early discharge from the hospital is assumed to be the ‘hotel cost’ of a hospital stay. These excess bed day costs were sourced from the 2017/18 NHS cost collection^12^ as this is the most recent source that reports the reference cost of excess bed days. These were inflated to 2021/2022 costs using the PSSRU inflation index.^9^ The cost for excess bed days for HF, calculated using the same HRG codes as for admission costs, is £338^10^ per patient, per day. This is based on the same HRG codes.

The number of ‘appointments’ for VW patients was monitored and included home visits (further characterized as routine or alert-related ‘rapid-response’ visits) and phone/virtual ‘calls’. All other care the patients received during the 30-day time horizon, such as emergency department attendances, hospital admissions from the VWs, and contact with NHS 111 was also monitored. *Table 1* provides a breakdown of the costs per appointment for each pathway. The cost of VW activity was calculated using the number of each activity, the time taken by staff to complete them, and the proportion of specific staff members and their banding levels who performed the tasks (*Table 2*). Staffing costs were calculated using the PSSRU Unit Costs of Health and Social Care 2023,^9^ were an average nurse works 1553 hours per year while a consultant works 2142 hours per year. The total cost for these roles includes wages/salary, salary oncosts, initial qualifications, ongoing training, and overheads such as management, administrative staff, estates staff, and non-staff costs, as well as capital overheads and travel costs. Using this ‘total cost per year’, the proportion of each role involved in performing a certain task, and their average working hours per year, we calculated the cost per minute to performing a task (*Table 2*). The costs of a broader range of resources to the NHS were calculated using the resource use and cost of those activities.

**Table 1:** Cost of appointments

Type of appointment	Cost, £	Source
Home visit	£102	NHS Cost Collection^10^
Emergency department attendance	£158	NHS Cost Collection^10^
111 contact	£11	Turner *et al.*^25^
Outpatient department appointment	£213	NHS Cost Collection^10^

**Table 2:** Time needed for VW activity (minutes) and staff banding performing the activity

Type of activity	Resource		Cost, £/min
Home visit/IV Furosemide @Home	Staff time (min)	45 min	
	Staff band	Bands 5 or 7	£1.02/min
Routine phone/virtual call	Staff time (min)	10	
	Staff band	Bands 7 or consultant	£1.79/min

SC inputs were sourced from a control group of patients who were admitted to an acute inpatient bed with the primary diagnosis of HF. The control group was propensity matched on a 1:1 ratio based on age, gender, and primary diagnosis by ICD 10 using ‘R Statistical Software’ (v4.1.2; R Core Team 2021). This included data on the proportion of control group patients readmitted to the hospital within 30 days, emergency department visits, and the number of NHS 111 calls. Additional inputs for SC have been sourced from academic literature where possible, with expert clinical opinion being used where an appropriate source was not identified.

Some costs were incurred at an aggregate level for all VWs; as a result, it has been assumed that costs are equally distributed between the VWs and their pathways. This is likely not reflective of how costs are distributed in other regions of the country but provides a baseline for comparison.

## Results

The average occupancy of the HFVW is approximately 88%. During the examination period, the HFVW supported a 36% absolute reduction in A&E activity and an 11% absolute reduction in NHS 111 activity. The model results for a cohort of 648 patients who were on VWs compared with SC are presented in *Table 3*. The total cost includes VW set-up cost, annual running costs of VWs, patient set-up costs, monitoring costs, appointment costs, POC tests and home intravenous therapy delivery, admission costs, and the cost savings from the reduced length of stay in hospital. The base case results show that the HF VW pathway is cost-saving, compared to SC with an incremental cost saving of £1135 per patient per episode (PPPE) in the first year.

**Table 3:** Total model results for HF VW pathway including set up costs

Summary results (including set up costs)	Virtual wards	Standard care	Incremental
Total annual costs per population	£467 524	£1 203 036	−£735 512
Total annual costs per patient	£721	£1857	−£1135

*Note*: Please note that totals may not match exactly due to rounding.

*Table 4* shows the total model results broken down by set-up, fixed, and variable costs for the VW pathway. Set-up costs are one-off costs in the model. Fixed costs are ones that do not vary with patient numbers in the model. This includes telehealth costs, overheads, non-pay costs, maintenance costs of monitoring technologies, licence costs, pharmacy, communications, clinical leadership, and programme manager costs. Variable costs vary with the number of patients in the model. These include staff costs, appointment costs, and inpatient costs.

**Table 4:** Total model results breakdown by cost type

Summary results breakdown	Virtual wards	Standard care	Incremental
Total one-off set-up costs	£4685	£0	£4685
Total on-going annual fixed costs	£308 113	£0	£308 113
Total annual variable costs	£154 727	£1 203 036	−£1 048 310

*Note*: Please note that totals may not match exactly due to rounding.

**Figure 2:**
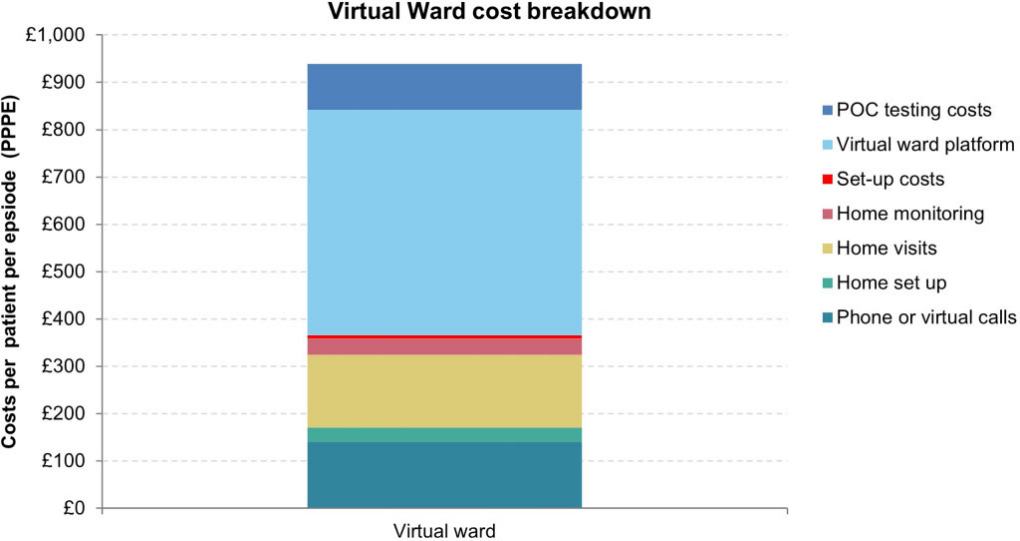
Direct costs breakdown per patient.

**Figure 3:**
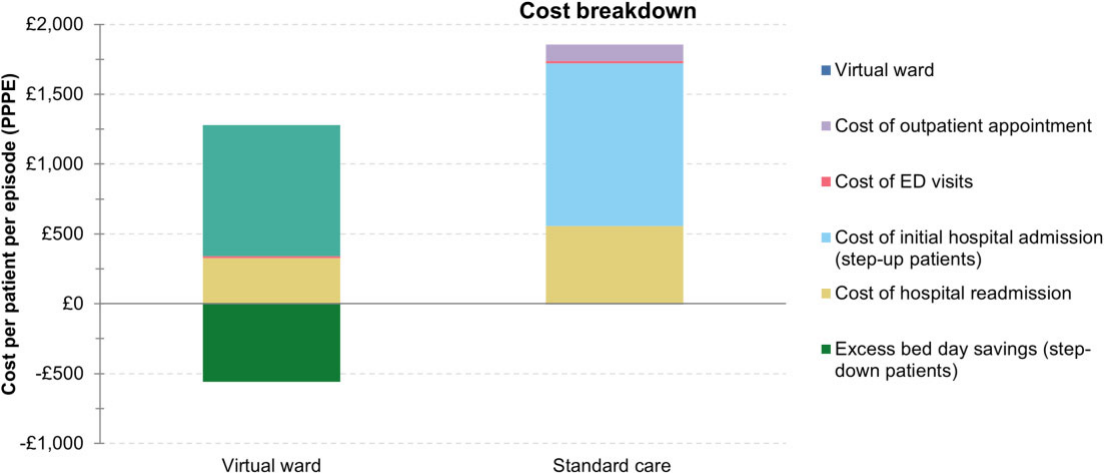
Indirect costs breakdown per patient.

*Table 5*, *Figures 2*, and *3* display a cost breakdown of costs per patient, which may allow the VW to identify areas where the VW has the potential to reduce resource use and costs. The VW platform includes costs for monitoring technologies, other non-pay costs, overhead costs, pharmacy, communications, clinical leadership, programme manager, and patient testing costs.

**Table 5:** Cost breakdown per patient for HF VW pathway

Direct costs	Virtual ward		
Virtual ward platform	£475		
Set-up costs	£7		
Home monitoring	£34		
POC testing costs	£98		
Home visits	£154		
Home set up	£30		
Phone or virtual calls	£140		
Total direct costs	£939		
**Indirect Costs**	**Virtual ward**	**Standard Care**	**Incremental ±**
Cost of outpatient appointment	£0	£117	−£117
Cost of ED visits	£14	£19	−£5
Cost of initial hospital admission (step-up patients)	£0	£1164	−£1164
Cost of hospital readmission	£326	£556	−£230
Excess bed day savings (step-down patients)	−£558	£0	−£558
111 contact	£1	£1	£0
Total indirect costs	−£218	£1857	−£2074
Total costs	£721	£1857	−£1135

*Note*: Please note that totals may not match exactly due to rounding.

### Sensitivity analysis

**Figure 4:**
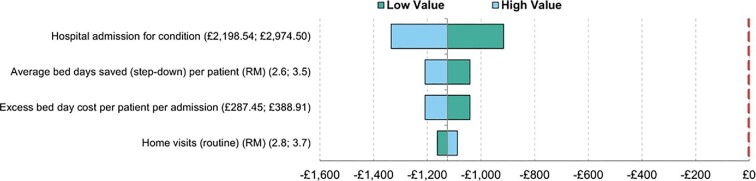
Tornado diagram for HFVW.

We included a deterministic sensitivity analysis (DSA) to investigate how sensitive the results are to any uncertainty in the key input parameters. Parameters were manually changed individually, and the results were analysed to determine to what extent the change has an impact on the output values. The range of variation of each parameter was assumed to be ±15% and the results are presented in a tornado diagram (*Figure 4*).

The horizontal bars are ordered so that those with the greatest spread (i.e. parameters to which the model output is most sensitive) are presented at the top of the diagram and those with the lowest spread at the bottom, with four of the most sensitive parameters shown in the figure. The red vertical line represents the breakeven point. Should any of the horizontal bars cross this threshold line, it indicates that variation in this input will change the direction of the results. In this example, there were four parameters tested using DSA: cost of hospital admission; step-down bed days saved; cost of excess bed days; and number of home visits. Varying these values by 15% either way had no impact on the result of the base case analysis that the VW was cost-saving. This means that potential uncertainty in these parameters has no impact on the economic conclusions and therefore, no implications for clinical management.

## Discussion

Our health economic analysis has demonstrated that our acute HF VW model provides a substantial net cost benefit of £1135 PPPE. This advantage remains evident despite the initial one-time setup expenses and ongoing costs encompassing a range of elements, including home visits (for intravenous diuretic therapy, scheduled or rapid-response visits), virtual or telephone consultations, POC testing, and the necessary equipment for monitoring patients at home.

The substantial net cost benefit of our acute HF VW model is primarily driven by several key factors. First, there is a marked reduction in hospital stay costs due to the shorter length of stay facilitated by ESD. Additionally, there is a significant decrease in the number of re-attendances to emergency departments and a reduction in hospital readmissions within 30 days. These factors collectively contribute to the overall cost savings. Separately, we have recently published our clinical outcomes from our HFVW, which demonstrate improvements in mortality rates and reduced readmission rates (sustained over a 1-year period).^13^ These clinical outcomes, combined with our demonstrated net cost benefit above, substantiate the cost-effectiveness of our HFVW model.

Moreover, our model predicts that with increased capacity and higher throughput in the HF VW program, the net cost benefit will likely grow further. Currently, our analysis indicates that the break-even point, where the costs of the VW and SC are equal, is achieved with only 185 patients annually. This threshold analysis underscores the efficiency and potential for scalability of the HF VW model in providing cost-effective care for our HF patients. Recent research has demonstrated a positive net financial benefit from a VW provision, with increasing cost benefit observed as the volume of admission rises. This analysis, performed in the South East of England^14^ has also shown that the use of VWs is associated with a positive impact on avoided NEL hospital activity—on average, 1 NEL admission ‘avoided’ was shown to be correlated with 2.5 VW admissions. Our findings align closely with these results, reinforcing the notion that VWs can be financially advantageous if both structured, and studied, appropriately.

Healthcare systems globally are facing financial challenges, requiring transformative changes in delivery models. The strategic implementation of a VW system is proposed as a supplementary innovative solution to help address these issues. VWs offer a dynamic and responsive approach to care, particularly beneficial for an ageing population and individuals with chronic conditions. By enabling efficient management of conditions at home, VWs reduce the need for hospital stays and have been shown to decrease hospital re-admission rates by 50% and the average length of hospital stays by 40%.^15,16^ Separate analysis of clinical outcomes from our HF VW has demonstrated a significant reduction in readmissions as well as mortality at 1, 3, 6, and 12 months. In addition, our data has also shown a reduction in adverse effects due to hospitalization such as hospital-acquired infections, delirium, falls, and adverse drug reactions.^13^

A recent meta-analysis^17^ assessed 24 randomized controlled trials (RCTs) of VWs (11 in patients with HF, three in patients with COPD, four in patients at high risk for readmission, and six in mixed patient populations) with over 10 000 patients. They found that in patients with HF, VWs were associated with fewer deaths and fewer readmissions. Similar associations were not seen in other diagnoses. However, across all studies, VWs were associated with fewer emergency department visits and shorter readmission lengths of stays. Additionally, three recent trials^18–20^ of VWs in a HF population found significant cost savings per patient.

With many healthcare systems facing physical capacity constraints in acute hospitals and ongoing limitations in capital availability, VWs can provide an innovative solution to help manage an increasing number of patients in their home environment and also facilitate effective discharge into other care settings. VWs also present an opportunity to create innovative workforce models, offering flexibility that could improve staff retention. For instance, VWs may allow clinicians to work from home, moving away from traditional expectations and contributing to more flexible workforce programmes. These service models also improve workforce experience, suggesting benefits for staff satisfaction.^21^

In addressing workforce challenges, VWs enable healthcare professionals to manage larger patient cohorts remotely, enhancing clinical team efficiency and addressing staffing shortages. The development of VW offers the potential to attract new staff, retain existing staff, and enhance integrated multidisciplinary team working across health and social care. Furthermore, VWs can be equipped with decision-support tools that are vital for patient recovery. These tools help analyse patient data to generate risk scores and guide clinical decision-making, such as the traffic-light systems for both patients and clinicians^13^ and by enabling early identification of high-risk patients with the use of validated risk scoring systems such as GWTG-HF.^22^ This proactive approach allows for targeted interventions to prevent complications and reduce readmission rates. The integration of VWs with existing healthcare systems ensures seamless information sharing, enhancing care continuity and quality by making comprehensive patient data accessible to the entire care team.

This analysis considers not just the budgetary costs allocated for the delivery of VW, but also the system-wide consequences, such as re-attendance to hospital and emergency department visits. The danger of only considering the budget provided for VWs and dividing it by the number of patients using VWs is that it does not consider the wider consequences of the intervention. For example, at face value, the cost per patient using a VW (if measured as the entire budget divided by the number of patients) may be lower than the unit cost of a bed day in the hospital. However, that scenario would not consider consequences such as the rate of admissions to hospital from VWs or readmissions to VWs, which could mean that VWs were not cost-saving. The wider consequences of VWs need to be considered to show the full costs and consequences of implementing the intervention.

In contrast to recent economic analysis literature,^23^ we have decided not to report the cost of a VW bed day in this analysis, as the temptation is for these to be compared with the corresponding hospital bed day costs. This can be both generalizing and misleading due to the complexity surrounding the question of what the appropriate comparator is. VWs should admit both step-up and step-down patients, which would incur different costs in the absence of VWs under the assumption all these patients would be in hospital otherwise. Therefore, the cost of a VW bed day could be compared with the cost of a bed day cost of a hospital admission for step-up patients.

As this is a study of ‘real-world patients,’ rather than a RCT, the judgment that all ‘step-up’ patients admitted to a VW would otherwise have been hospitalized was made by the VW team based on the assessment of clinical acuity, and this assumption may not have been free of inherent biases (such as treatment selection bias) or errors in clinical judgment. The same principle applies to step-down patients, where the assumption is that they would have stayed in the hospital for the corresponding duration when managed on a VW. These assumptions, while necessary, may not be free from subjectivity. It is important to recognize that these limitations are not exclusive to VW models; subjective clinical judgment also plays a role in standard care, particularly in decisions around hospitalization and discharge.

Patient, family, and carer experiences significantly improve with VWs, aligning with expectations for personalized, patient-centred, and accessible healthcare.^24^ VWs enable patients to receive care at home, reducing stress and inconvenience while enhancing communication and coordination for a more engaged and informed care process. Caring for people in their own homes supports recovery, autonomy, and choice, with studies showing that VW models reduce AED attendance and unplanned hospital admissions by meeting patient needs earlier. Research indicates a high likelihood of patients recommending VW stays to family and friends, with a 99.5% satisfaction rate within our patient cohort.

### Limitations

Although the ICB has made significant progress with the VW initiative, it is still evolving. Therefore, the results of this baseline analysis should not be viewed as a definitive conclusion on the cost-effectiveness of individual VWs elsewhere. The reported results represent a specific point in time, but it is important to note the dynamic nature of VWs, where inputs can vary week by week. Continuous monitoring of VWs and their system-wide benefits as they develop, is therefore essential.

Second, the analysis does not account for the relative acuity of patients. This is an important consideration because acuity can vary across different VWs and within VWs over time or during different times of the year. Furthermore, we have employed the assumption that all step-up patients would have been admitted to the hospital in the absence of a VW.

Third, while *this* analysis is comprehensive in terms of resource use, it does not consider any changes in clinical health outcomes for people receiving VW care compared with SC. Therefore, we have employed the term ‘net cost benefit’ to describe our findings, which more accurately aligns with definitions from a health economics perspective.

### Conclusion

Our health economic analysis has demonstrated that our HF VW model provides a significant net cost benefit of £1135 PPPE. This advantage remains evident despite the initial one-time setup expenses and ongoing costs encompassing a range of elements, including home visits (for intravenous diuretic therapy, scheduled or rapid-response visits), virtual or telephone consultations, POC testing, and the necessary equipment for monitoring patients at home.

From a health economic perspective, accurately using the term ‘cost-effective’ necessitates evaluating both the net cost benefits and the clinical outcomes. This dual consideration ensures that any intervention, such as a VW, is not cost incurring and also confers improved clinical outcomes. We have also published recently regarding the beneficial clinical outcomes due to acute HF VWs in terms of reduced readmissions and mortality.^13^ Therefore, we assert that our HFVW is cost-effective, supported both by thorough economic analysis and a clinical evaluation.

## Data Availability

Data available on request—The data underlying this article will be shared on reasonable request to the corresponding author.
